# Genetic Tracing of Ca_v_3.2 T-Type Calcium Channel Expression in the Peripheral Nervous System

**DOI:** 10.3389/fnmol.2017.00070

**Published:** 2017-03-15

**Authors:** Yinth A. Bernal Sierra, Julia Haseleu, Alexey Kozlenkov, Valérie Bégay, Gary R. Lewin

**Affiliations:** Department of Neuroscience, Max Delbrück Center for Molecular Medicine in the Helmholtz Association and Charité – Universitätsmedizin BerlinBerlin, Germany

**Keywords:** Ca_v_3.2, genetic tracing, sensory neuron progenitors, D-hair receptors, hairy skin, slowly-adapting mechanoreceptors, muscle nociceptors, spinal cord

## Abstract

Characterizing the distinct functions of the T-type ion channel subunits Ca_v_3.1, 3.2 or 3.3 has proven difficult due to their highly conserved amino-acid sequences and the lack of pharmacological blockers specific for each subunit. To precisely determine the expression pattern of the Ca_v_3.2 channel in the nervous system we generated two knock-in mouse strains that express EGFP or Cre recombinase under the control of the Ca_v_3.2 gene promoter. We show that in the brains of these animals, the Ca_v_3.2 channel is predominantly expressed in the dentate gyrus of the hippocampus. In the peripheral nervous system, the activation of the promoter starts at E9.5 in neural crest cells that will give rise to dorsal root ganglia (DRG) neurons, but not sympathetic neurons. As development progresses the number of DRG cells expressing the Ca_v_3.2 channel reaches around 7% of the DRG at E16.5, and remains constant until E18.5. Characterization of sensory neuron subpopulations at E18.5 showed that EGFP^+^ cells are a heterogeneous population consisting mainly of TrkB^+^ and TrkC^+^ cells, while only a small percentage of DRG cells were TrkA^+^. Genetic tracing of the sensory nerve end-organ innervation of the skin showed that the activity of the Ca_v_3.2 channel promoter in sensory progenitors marks many mechanoreceptor and nociceptor endings, but spares slowly adapting mechanoreceptors with endings associated with Merkel cells. Our genetic analysis reveals for the first time that progenitors that express the Ca_v_3.2 T-type calcium channel, defines a sensory specific lineage that populates a large proportion of the DRG. Using our Ca_v_3.2-Cre mice together with AAV viruses containing a conditional fluorescent reporter (tdTomato) we could also show that Cre expression is largely restricted to two functionally distinct sensory neuron types in the adult ganglia. Ca_v_3.2 positive neurons innervating the skin were found to only form lanceolate endings on hair follicles and are probably identical to D-hair receptors. A second population of nociceptive sensory neurons expressing the Ca_v_3.2 gene was found to be positive for the calcitonin-gene related peptide but these neurons are deep tissue nociceptors that do not innervate the skin.

## Introduction

T-type calcium channels belong to the family of low voltage activated Ca^2+^ (LVA) channels encoded by three α1 subunit genes: α1G (Ca_v_3.1), α1H (Ca_v_3.2), and α1I (Ca_v_3.3) (Perez-Reyes, 1999). LVA channels are involved in cell excitability due to their low voltage threshold for activation (Cain and Snutch, 2010), and shape neuronal firing patterns because of their unique gating properties (Fox et al., 1987; Perez-Reyes et al., 1998; Vassort et al., 2006; Powell et al., 2009; Isope et al., 2012). Unfortunately, determination of the individual contribution of each T-type subunit to these physiological processes has been hampered by the lack of specific subtype pharmacological blockers, and the fact that in native systems more than one T-type channel is often expressed (Perez-Reyes, 2003). Also, their highly conserved primary amino-acid sequence has made the production of antibodies specific for individual subunits extremely challenging (Cribbs et al., 1998). Nevertheless, the expression and distribution of the mRNA transcripts and protein for each subunit in the brain and dorsal root ganglia (DRGs) of rats has been described (Talley et al., 1999; McKay et al., 2006).

In order to separate the functions of these three different channel subunits, an important first step is to determine exactly where and when each individual subunit is expressed. We are interested in the Ca_v_3.2 ion channel’s role in the somatosensory system. Some studies show a role for this subunit in peripheral nociception (Bourinet et al., 2005; Choi et al., 2006; Wen et al., 2006), while others have emphasized a role of this channel in the physiology of an ultrasensitive subpopulation of mechanoreceptors in the DRG, the D-hair receptors (Shin et al., 2003; Dubreuil et al., 2004; Wang and Lewin, 2011; Lechner and Lewin, 2013; François et al., 2015) and recently also C-fiber low-threshold mechanoreceptors (C-LTMRs) (François et al., 2015). To resolve issues of where and when Ca_v_3.2 channels are expressed we used gene targeting to generate two knock-in strains: the Ca_v_3.2*^EGFP^* and the Ca_v_3.2*^Cre^*. The first expresses EGFP and the second Cre recombinase under the control of the Ca_v_3.2 gene promoter. The Ca_v_3.2*^EGFP^* strain allowed us to examine the spatiotemporal expression of Ca_v_3.2 ion channels since detection of EGFP represents a measure of Ca_v_3.2 gene promotor activity. We determined the onset of the Ca_v_3.2 gene expression during development in the nervous system and characterized the type of neurons expressing the Ca_v_3.2 channel in the DRG at E18.5 and in the brain of adult mice. The Ca_v_3.2*^Cre^* strain was used in conjunction with the Tau*^mGFP^* reporter mouse which is specific for the nervous system or the Rosa26*^LacZ^* strain which is a ubiquitous reporter line. In this way we could determine cell types that had expressed the Ca_v_3.2 ion channel at any point throughout development. Moreover, the reporter line used here also allowed us to visualize those cells that express or had expressed Ca_v_3.2 both *in vitro* and *in vivo*. Using this approach, we observed that the expression of the Ca_v_3.2 gene was absent in certain neural crest lineages giving rise to slowly adapting mechanoreceptors (SAMs) in the sensory lineage. We also show that viral-based gene transfer in adult Ca_v_3.2*^Cre^* mice is a powerful tool to label specific sensory receptor subtypes.

## Materials and Methods

This study was carried out in accordance with the recommendations of the German animal welfare laws and protocols for euthanasia and virus injections were approved by German federal authorities (State of Berlin).

### Generation of Knock-In Mice

To generate the Ca_v_3.2*^EGFP^* and the Ca_v_3.2*^Cre^* knock-in mice, the sequence of EGFP containing the membrane targeting signal GAP43 (Mosevitsky, 2005), or Cre was inserted after the start codon of the Ca_v_3.2 gene located at the beginning of the second exon (Supplementary Figures 1A,B). The insertion of either the EGFP or Cre cassettes should produce a null mutation of the Ca_v_3.2 gene as the EGFP or Cre cassette will be transcribed ending with a stop codon and a poly-A signal (Supplementary Figures 1A,B). A large number of splice variants of the human Ca_v_3.2 gene have been described (Zhong et al., 2006) but all these splice variants use the same start codon and it is therefore unlikely that transcripts exist with in frame alternative start codons. Examination of the mouse Ca_v_3.2 gene sequence showed that all internal ATG codons would only exist as transcripts encoding peptides unrelated to Ca_v_3.2. Using quantitative real time PCR we also could not detect mature Ca_v_3.2 transcripts from the brains of Ca_v_3.2*^EGFP^* homozygous knock-in mice (data not shown). Targeting vectors to insert EGFP or Cre into the Ca_v_3.2 mouse gene were made using the recombineering approach (Liu et al., 2003). First, mini-targeting vectors were produced. Two homology arms (400 bp each) containing the sequence flanking the targeting site in the Ca_v_3.2 allele were PCR amplified, and cloned left and right of the *EGFP*-*lox*P-*Neo-lox*P cassette or the *Cre*-*FRT-Neo-FRT* cassette. Subsequently, to release the cassette flanked with the homology arms, each mini-targeting vector was digested with *Hind*III*Bst*XI or *Sac*I restriction enzymes respectively, and the fragment was gel purified. Each of the gel purified fragments were co-transfected with the pSKCa_v_3.2DTA plasmid into temperature activated EL350 cells by electroporation. The pSKCa_v_3.2DTA vector contained a 9.5 kb fragment of the Ca_v_3.2 sequence containing the exon 2 of the gene. The *Red* genes (λ-encoded genetic recombination protein machinery) activated by heat shock in EL350 cells allowed the homologous recombination of the DNA and the generation of the targeting vectors pCa_v_3.2*^EGFP^* and pCa_v_3.2*^Cre^*. After electroporation of the EL350 cells, 400 μL of SOC medium was added and the cells were grown at 32°C for 1 h with shaking (200 rpm). Cells were spread on plates with appropriate antibiotics. The positive clones were confirmed by sequencing and used for gene targeting in mouse embryonic stem (ES) cells. The pCa_v_3.2*^EGFP^* or pCa_v_3.2*^Cre^* targeting vector was linearized with *Swa*I and electroporated into 129/Sv ES cells. Homologous recombinants were identified by Southern blot analysis of *BamH*I (Supplementary Figure 1A) or *EcoR*V digested genomic DNA (Supplementary Figure 1B) respectively, using an external 5′ end probe. Confirmation of correct insertion of the cassette at the 3′ end of the targeting site as well as determination of only one insertion event in the mouse genome was made using Southern blot analysis (data not shown). A minimum of three correctly targeted ES clones were selected and used to produce germ line chimeras by blastocyst injection. Male chimeras were mated to C57BL/6N mice to obtain the first filial (F_1_) generation. Further generations were also mated to C57BL/6N mice and the offspring carrying the targeted allele were identified by PCR of tail DNA with primers that amplified the EGFP or Cre sequence. Ca_v_3.2*^Cre^* mice were bred with Flpe deleter [Tg(ACTFLPe)9205Dym] mice (Rodríguez et al., 2000) while Ca_v_3.2*^EGFP^* mice were bred with Cre deleter [Tg(CMV-cre)1Cgn] mice to remove the Neo cassette (Schwenk et al., 1995). Homozygous mice of both strains were viable, fertile, and born at a Mendelian ratio. Genotyping of the Ca_v_3.2*^EGFP^* knock-in mice was performed using the forward primer TCA CCG CCT GCC CCT CTC TCC G and the reverse primer GTA CGC AGA GGA GTC CCA CA, and of the Ca_v_3.2*^Cre^* knock-in mice using the forward primer TAA CTA CCT GTT TTG CCG GG and the reverse primer as shown above. The wild type allele was amplified using the forward primer TCA CCG CCT GCC CCT CTC TCC G and the reverse primer as shown above. Homozygous Ca_v_3.2*^Cre^* mice were bred with *Tau^mGFP^* (*Tau*-*lox*P-STOP-*lox*P-*mGFP*-IRES-NLS-*LacZ*-pA) (Hippenmeyer et al., 2005) or Rosa26*^LacZ^* (R26R-*lox*P-PKG*neo*4xpA-*lox*P-*LacZ*-pA) (Soriano, 1999) mice. In the *Tau^mGFP^* reporter mouse, the expression of myristylated GFP (*mGFP*) and β-gal is activated in neurons once the stop signal flanked by two *lox*P sites is removed by CRE recombinase. The mGFP is then targeted to the membrane while β-gal, which is tagged with a nuclear localization signal (NLS), is nuclear (Hippenmeyer et al., 2005). In the Rosa26*^LacZ^* reporter line, cytoplasmic β-gal is ubiquitously expressed in embryos and adult mice upon commencement of activity of CRE recombinase (Soriano, 1999).

### Whole Mount Staining of β-gal Expressing Embryos

After mice were bred, the presence of a vaginal plug was considered as embryonic day 0.5 (E0.5). Timed pregnant mice were euthanized by cervical dislocation, mouse embryos were dissected in 0.1 M ice-cold phosphate-buffered saline (PBS), and extra-embryonic membranes were removed. Embryos older than E15.5 were de-skinned and eviscerated to facilitate penetration of the X-gal staining solution. Prepared embryos were fixed for 2–6 h in 0.5% glutaraldehyde and rinsed thoroughly in ice-cold PBS. The embryos were incubated overnight at 37°C protected from light in freshly prepared X-gal buffer (35 mM potassium ferrocyanide, 35 mM potassium ferricyanide, 2 mM MgCl_2_, 0.02% Nonidet P-40, 0.01% Na deoxycholate in PBS; before use, 38 ml of the buffer was mixed with 2 ml of 20 mg/ml X-gal dissolved in dimethylformamide). After staining, the embryos were washed in PBS to remove the rest of the X-gal buffer, and fixed in 4% PFA overnight at 4°C. Embryos were cleared as previously described (Schatz et al., 2005). Each incubation step lasted from 4 to 7 days. Cleared embryos were stored in 100% glycerol and images were acquired with a Leica M165C stereomicroscope.

### Immunofluorescence

Four to ten week old mice were anesthetized and transcardially perfused with 0.1 M PBS followed by 4% paraformaldehyde (PFA). Skin and brain were post-fixed in 4% PFA for 2 h at 4°C and spinal cord for 30 min. After fixation tissues were washed three times with PBS and dehydrated by incubating them overnight at 4°C in solutions of 10, 20, and 30% of sucrose. DRGs, spinal cords, muscle tissue, and skin were embedded in Tissue-Tek O.C.T. Compound (Sakura Finetek, Netherlands), and frozen on dry ice while brains were frozen in dry ice-cooled isopentane for a maximum of 20 s. Tissues were stored at -80°C. Frozen embedded tissues were sectioned on a Cryostat (Leica CM3050S), transferred to slides, and dried at room temperature. Section thickness was 18 μm for DRGs and muscle, 16 μm for brain, and 40–50 μm for skin. Mouse embryos at stages E9.5, E13.5, E16.5, and E18.5 were dissected from euthanized pregnant mice, fixed in 4% PFA for 2–6 h at 4°C, embedded in O.C.T., and processed further in the same manner as adult tissues. Embryos older than E15.5 were de-skinned and eviscerated.

Slides were washed in tris-buffered saline (TBS) and incubated for 2 h at room temperature in a pre-incubation buffer containing 1% BSA and 0.1% Triton-X-100. Subsequently, the slides were incubated with the first antibody diluted in incubation buffer (TBS buffer with 5% of appropriate normal serum and 0.05% Triton-X-100) overnight at 4°C. The next day, sections were washed three times for 10 min each with TBS buffer, followed by incubation with secondary antibody diluted in incubation buffer for 2 h at room temperature. For labeling of non-peptidergic nociceptive neurons, isolectin B4 conjugated with Alexa Fluor 488 (IB4; Molecular Probes, Cat# I21411, RRID: AB_2314662) was added to the secondary antibody solution at a concentration of 10 μg/ml. The sections were then washed three times with TBS for 10 min each, and Hoechst dye (Sigma-Aldrich) was added at a 1:10,000 dilution in the last wash. Slides were washed for 5 min with double-distilled water and mounted. Images were acquired with a confocal microscope (LSM 700; Carl Zeiss). Primary antibodies used were: goat antiTrkB (R&D Systems, Cat# AF1494, RRID: AB_2155264) 1:2000, goat anti-TrkC (R&D Systems, Cat# AF1404, RRID: AB_2155264) 1:2000, rabbit antiTrkA (a gift from Prof. Louis F. Reichardt, UCSF) 1:1000, sheep anti-Tyrosine Hydroxylase (Millipore, Cat# AB1542, RRID: AB_90755) 1:400, rabbit anti-CGRP (Sigma-Aldrich, Cat# C8198, RRID: AB_259091) 1:1000, mouse anti-CK20 (Dako, Cat# M7019, RRID: AB_2133718) 1:200, chicken antiGFP (Abcam, Cat# ab13970, RRID: AB_300798) 1:1000, rabbit antiGFP (Abcam, Cat# ab6556, RRID: AB_305564) 1:1000, chicken antiNF200 (Abcam, Cat# ab72996, RRID: AB_2149618) 1:3000, rabbit antiS100 (Dako, Cat# Z0311, RRID: AB_10013383) 1:1000, chicken antiβgal (Abcam, Cat# ab9361, RRID: AB_307210) 1:1000. Secondary antibodies of the relevant species and coupled to Alexa fluorophores (488, 633, 647), Cy2, or Cy3 were used at a 1:500 or 1:1000 dilution and purchased from Invitrogen or Jackson ImmunoResearch.

### Viral Particle Injections

Four to six week old heterozygous Ca_v_3.2*^Cre^* mice were anesthetized by an intraperitoneal injection of ketamine (100 mg/kg) and xylazine (10 mg/kg). The sciatic or saphenous nerve were exposed and 2 μl of AAV-flex-tdTomato (AV2/9.CAG.FLEX.tdTomato.WPRE.bGH; Penn Vector Core, Philadelphia, PA, USA) with a titer of 3.71 E+12 were slowly injected using a pulled glass capillary attached to a Hamilton microliter syringe. After the injection, the capillary was left in place for additional three min.

For intrathecal injections, a dorsal laminectomy of one lumbar vertebra was performed. Using a pulled glass capillary, the dura was punctured and 2 μl of AAV-flex-tdTomato (Penn Vector Core, Philadelphia, PA, USA) were slowly injected. Again, after the injection the capillary was left in place for additional three min. Four weeks post-injection, the animals were transcardially perfused.

### Immunostaining of Thick Skin Slices

Dissected skin was post-fixed in 4% PFA overnight. Subsequently, the skin was washed three times in PBS at room temperature for 10 min each and embedded in 3% low-melting agarose. Using a vibratome, the skin was cut into 100 μm thick transverse slices. Prior to antibody incubation, the skin slices were washed in blocking solution (5% normal serum, 0.1% Triton X-100 in 0.1 M PBS) at 4°C for 1 h. Skin slices were incubated with primary antibodies diluted in blocking solution at 4°C for 24 h. Next, the slices were washed three times in 0.1 M PBS for 10 min each at room temperature and incubated with secondary antibodies diluted in blocking solution at 4°C for 24 h. Primary antibodies used were: chicken antiNF200 (Abcam, Cat# ab72996, RRID: AB_2149618) 1:2000, rabbit antiPGP9.5 (Dako, Cat# Z5116, RRID: AB_2622233) 1:500, rabbit antiS100 (Dako, Cat# Z0311, RRID: AB_10013383) 1:1000, guinea pig antiCK20 (Acris Antibodies GmbH, Cat# BP5080, RRID: AB_979693) 1:1000, rabbit antiTrkB (R&D Systems, Cat# AF1494, RRID: AB_2155264) 1:200. Secondary antibodies (Invitrogen) were coupled to Alexa Fluor dyes (488, 647) and used at a dilution of 1:500.

After completed immunostaining, skin slices were washed three times in 0.1 M PBS for 10 min each and immersed in ascending concentration series of 2′2-thiodiethanol (TDE; Sigma-Aldrich) diluted in 0.1 M PBS for 12 h each at room temperature. The concentration steps were 10, 25, 50, and 97% TDE. All incubation steps were performed under agitation and in the dark.

### Two-Photon Imaging of Cleared Spinal Cord Tissue

Unsectioned spinal cord tissue was dehydrated and de-lipidated in 50, 80, and 100% THF for 30 min each at room temperature. Subsequently, the tissue was immersed in ascending concentrations steps of TDE diluted with 0.1 M PBS for 12 h each at room temperature. The concentration steps were: 10, 25, 50, and 97%. All incubation steps were performed under agitation and in the dark.

For two-photon imaging, the tissue was mounted in 97% TDE using Press-to-Seal Silicone Isolators (Electron Microscopy Sciences).

### RNA Isolation and Quantitative Real-Time PCR

Total RNA was isolated from tissues (DRGs and brain) with TRIzol reagent according to the manufacturer’s recommendations (Thermo Fisher Scientific). Freshly dissected tissues were immediately transferred into Eppendorf tubes containing an appropriate volume of TRIzol (normally 1 ml per 100 mg of tissue), and the samples were homogenized in Precellys 24-Dual homogenizer using a two-step program. Each step homogenized the sample with a 3D motion speed of 5000 rpm for 25 s. After isolation, total RNA was washed with 75% ethanol, air-dried, and dissolved in 30 μl of RNAse-free water. To eliminate contamination of the RNA with DNA, the samples were incubated with DNAase for 30 min at 37°C using TURBO DNA-free kit (Thermo Fisher Scientific). Two μg of total RNA was used for cDNA synthesis using SuperScript III Reverse Transcriptase (Invitrogen) according to the manufacturer’s protocol using random primers and Oligo-dT. For real time qPCR, 10 μL final reaction volume was used. One μl cDNA or water was added to a mix containing: 0.4 μM primers, final concentration, forward primer TGGTCCTGCTGGAGTTCG, reverse primer CTTGTACAGCTCGTCCATGC, 5 μL of universal PCR master mix from Roche and 0.1 μL of Probe No. 70 from the mouse Universal Probe Library from Roche. To make an absolute quantification of EGFP mRNA, the standard curve was prepared using serial dilutions of pEGFP-N3 plasmid ranging from 5 × 10^1^ to 5 × 10^8^ moles per reaction. Each sample and every single point of the standard curve were quantified in triplicate. The samples and the standard curve were set up in 384 well-plates and run on an ABI Prism 7900 sequence detection system using a two-step protocol. The program starts with denaturation at 95°C for 10 min, the first step 95°C for 10 s followed by a second step at 60°C for 1 min, 40 cycles. Cycle threshold (Ct) values were set manually.

### SDS-PAGE and Western Blot Analysis

Dorsal root ganglia or brain were isolated and immediately immersed in RIPA lysis buffer supplemented with EDTA-free protease inhibitor cocktail tablets (Roche). Samples were homogenized in a Precellys 24-Dual homogenizer using a two-step program; 5000 rpm for 25 s each step. Then, the samples were incubated for 20–30 min with rotation and subsequently centrifuged for 20 min at 16000 *g*. The supernatant was transferred into a new tube and centrifuged for 30 more min. All the steps were performed at 4°C. Protein concentrations were determined using the Pierce BCA Protein Assay Kit. 20 μg of protein were mixed with loading buffer (12.5 mM Tris base, 50 mM EDTA, 1% SDS, 1% β-Mercaptoethanol and 0.005 g/ml Bromophenol blue) and denatured for 5 min at 95°C. Cooled samples were then loaded on a 13% SDS gel and proteins separated in Tris-glycin buffer at 120–150 V on a Mini-PROTEAN III system (Bio-Rad). Proteins separated on the SDS-PAGE gel were blotted on a nitrocellulose membrane using a wet-blotting Mini Trans-Blot system (Bio-Rad) according to the manufacturer’s instructions. The membrane was rinsed with TBS buffer and incubated in blocking buffer for non-specific binding of antibody with shaking for 1 h at room temperature. The blocked membrane was washed twice for 5 min each in TBS-0.5% Tween buffer followed by incubation with the primary antibody diluted in TBS-0.5% Tween buffer for 1 h with shaking. Non-bound antibody was washed with TBS-0.5% Tween buffer (three times for 5 min each) and the membrane was incubated in the secondary peroxidase-conjugated antibody pre-diluted in TBS-0.5% Tween buffer. The membrane was washed again in the same manner and developed with Amersham^TM^ ECL plus Western blot detection reagent. The chemiluminescence signal was detected after exposure (15 min) of the membranes to ECL films (GE Healthcare).

### DRG Cell Culture

Adult mice were euthanized by cervical dislocation. DRGs were dissected and collected in a 1.5 ml tube in PBS on ice. Ganglia were washed once with PBS before incubation with 1 μg/ml Collagenase Type-IV (Sigma) at 37°C for 25 min followed by centrifugation at 170 ×*g* for 3 min. The supernatant was removed and DRGs were incubated with 0.05% Trypsin-EDTA (Invitrogen) in 1 ml PBS at 37°C for 20 min. The supernatant was removed and 1 ml D-MEM/F12 medium was added. The medium contained 10% heat-inactivated horse serum (Invitrogen), 20 mM glutamine, 0.8% glucose, 100 U penicillin and 100 mg/ml streptomycin (Invitrogen). The suspension was passed through a 23G injection needle to dissociate the cells, and centrifuged at 170 × *g* for 4 min. The supernatant was removed and cells were resuspended in 1 ml culture medium. Cells were plated on laminin-coated coverslips (20 μg/ml), pre-coated with Poly-L-lysine (200 mg/ml). About 1000–2000 cells were plated per coverslip. Cells were maintained for 24 h at 37°C in 5% CO_2_.

### Electrophysiology

Whole-cell recordings were made at room temperature (20–24°C) from DRG cell cultures. Fire-polished patch pipettes with a resistance of 4–6 MΩ were used. The electrode was filled with patch clamp buffer containing: 110 mM KCl, 10 mM Na^+^, 1 mM MgCl_2_, 1 mM EGTA, 10 mM HEPES (pH 7.3). The extracellular solution used contained: 140 mM NaCl, 1 mM MgCl_2_, 2 mM CaCl_2_, 4 mM KCl, 4 mM glucose, 10 mM HEPES (pH 7.3). Voltage measurements were acquired and amplified using an EPC-10 amplifier sampled at 10 kHz (HEKA). Pipette and membrane capacitance were compensated using the auto function of Patchmaster and series resistance was compensated by 70% to minimize voltage errors. Action potentials (APs) were recorded in the current clamp configuration (bridge mode) by injecting 80 ms duration square wave current pulses with amplitude increments of 80 pA. Recordings were analyzed with the Patchmaster and Fitmaster software (HEKA).

## Results

**FIGURE 1:**
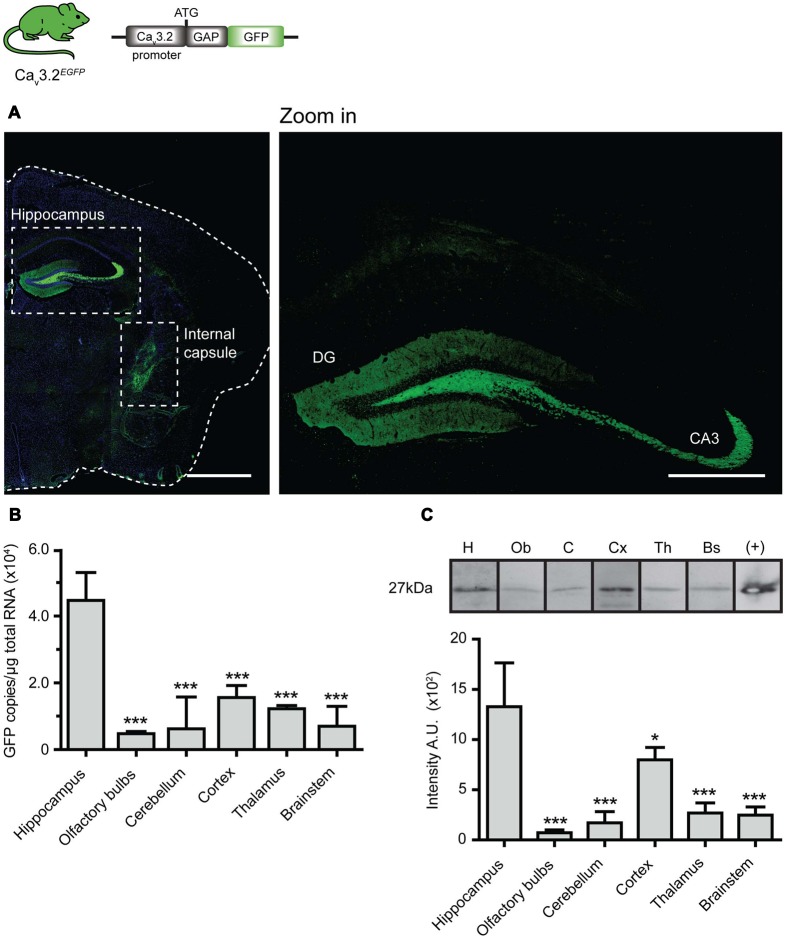
**EGFP expression pattern in brain of Ca_v_3.2*^EGFP^* knock-in mice. (A)** EGFP immunostaining of brain coronal sections of Ca_v_3.2^GFP^ mouse. Positive staining is observed only in axons of the hippocampus (also see zoom in) and in the internal capsule. Scale bar: 1 mm; scale bar in the zoom in panel: 500 μm. **(B)** Quantification of GFP mRNA in different regions of the brain by qPCR. The amount of EGFP molecules per μg of total RNA used for reverse transcription was calculated using a standard curve constructed with consecutive dilutions of the pEGFP-N3 plasmid (Ct vs. log[EGFP]). The amount of EGFP molecules in the hippocampus was compared to those from other regions in the brain. ^∗∗∗^*P* ≤ 0.001, *n* = 3, Dunnet’s multiple comparison test. Data presented as mean + SD. **(C)** Lower panel shows representative Western blots of different brain regions with the GFP protein band at 27 kDa. Note that bands were imaged from the same Western blot but the lane order was changed for presentation purposes. Protein extract from HEK cells transfected with pEGFP-N3 was used as a positive control. The bar graph illustrates the densitometry quantification of EGFP in brain. Arbitrary units (AUs) from the hippocampus were compared to those from the other regions of the brain. ^∗^*P* ≤ 0.05; ^∗∗∗^*P* ≤ 0.001, *n* = 3, Dunnet’s multiple comparison test. Data presented as mean ± SD.

We generated the Ca_v_3.2*^EGFP^* knock-in mouse in order to unambiguously visualize cells expressing the Ca_v_3.2 channel. We observed strong EGFP staining in the brains of Ca_v_3.2*^EGFP^* adult animals, e.g., in the dentate gyrus (DG) of the hippocampus (**Figure 1A** and zoom in), and in axons located in the internal capsule (**Figure 1A**). The GAP43 membrane targeting signal attached to the EGFP molecule explains why the majority of the staining is observed in mossy fiber axons coming from dentate granule neurons (Mosevitsky, 2005). The positive staining observed in the internal capsule is presumably derived from the ascending or descending axons that run between the cerebral cortex and the pyramids of the medulla. Previous reports demonstrated that the Ca_v_3.2 channel is also expressed in other brain regions of rats such as the olfactory system, insidium griseum in the basal forebrain, amygdala, hypothalamus, reticular thalamic nucleus and layer V of the neocortex (Talley et al., 1999; McRory et al., 2001; Chen et al., 2010). We considered the possibility that a low expression level of Ca_v_3.2 in these cells made the immunodetection of EGFP difficult in these brain regions. To test this idea, we used real time qPCR and Western blotting to detect the levels of EGFP mRNA and protein present in these tissues in our knock-in mice (**Figures 1B,C**). Real time qPCR from total RNA of P0 Ca_v_3.2*^EGFP^* homozygous animals showed EGFP expression in hippocampus, olfactory bulbs, cerebellum, neocortex, thalamus, and brainstem (**Figure 1B**). This experiment revealed that the absolute level of EGFP molecules present in the hippocampus was, on average, higher compared to the other brain regions. We could also detect EGFP protein in all the tested brain regions using Western blotting; subsequent densitometry analysis of the blots also showed higher EGFP levels in the hippocampus compared to other brain regions (**Figure 1C**). It should be noted that all the analysis of Ca_v_3.2*^EGFP^* were carried out on homozygous mice as we had difficulty detecting EGFP in Ca_v_3.2*^EGFP/+^* mice. Homozygous Ca_v_3.2*^EGFP^* mice were likely null for Ca_v_3.2 channel expression as we noted that some mice showed premature death which is consistent with the presence of cardiac disease described in mice with a targeted deletion of this gene (Chen et al., 2003).

**FIGURE 2:**
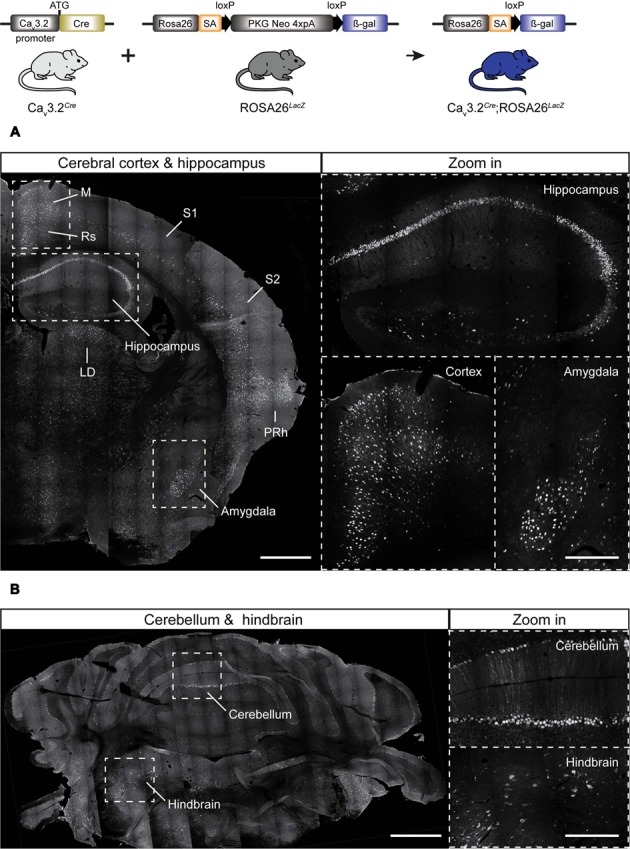
**Cre activation in the brain of Ca_v_3.2*^Cre^*; *Rosa26^LacZ^* mice. (A)** Coronal section of Ca_v_3.2*^Cre^*;Rosa26*^LacZ^* adult mouse brain stained using antibody against β-gal. Dashed lines indicate brain regions that are shown as a zoom in: hippocampus, cortex, and amygdala. In these regions, dense and intense βgal signal was observed. **(B)** Coronal sections of cerebellum and hindbrain. Notice that not every single Purkinje cell of the cerebellum was positive (zoom in). Positive staining for β-gal in some cells of the hindbrain are shown in a zoom in. Scale bars: 600 μm; scale bars in the zoom in panels: 300 μm. M, motor cortex; Rs, retrosplenial cortex; S1, primary somatosensory cortex; S2, secondary somatosensory cortex; LD, laterodorsal thalamic nucleus; PRh, perirhinal cortex.

Considering that the Ca_v_3.2*^Cre^* strain allows a more sensitive detection of the Ca_v_3.2 channel promoter activity when crossed with a reporter line, we next examined adult Ca_v_3.2*^Cre^*;Rosa26*^LacZ^* mice. In these mice, β-gal is expressed in cells in which Cre is driven by the Ca_v_3.2 promoter. In the brains from Ca_v_3.2*^Cre^*;Rosa26*^LacZ^* mice we observed β-gal positive cells in the cortex, hippocampus, cerebellum, and brainstem (**Figure 2A**). In the hippocampus, positive β-gal cells were detected in the pyramidal layers of fields CA1, CA2, and CA3. In addition, scattered cells were observed in the hilus of the DG (**Figure 2A** and zoom in). In the cortex, β-gal positive cells were observed in layers I–VI in the retrosplenial, somatomotor and perirhinal areas, and in layer V in the somatosensory areas (**Figure 2A** and zoom in). Strong staining was also observed in cells of the amygdala in the striatum (**Figure 2A** and zoom in), in the brainstem some β-gal^+^ cells were found in the latero-dorsal thalamic nucleus (**Figure 2A**), and in the medulla in the hindbrain (**Figure 2B** and zoom in). In the cerebellum, the expression of β-gal seems to be restricted to only a few of the Purkinje cells within the cerebellar cortex (**Figure 2B** and zoom in).

**FIGURE 3:**
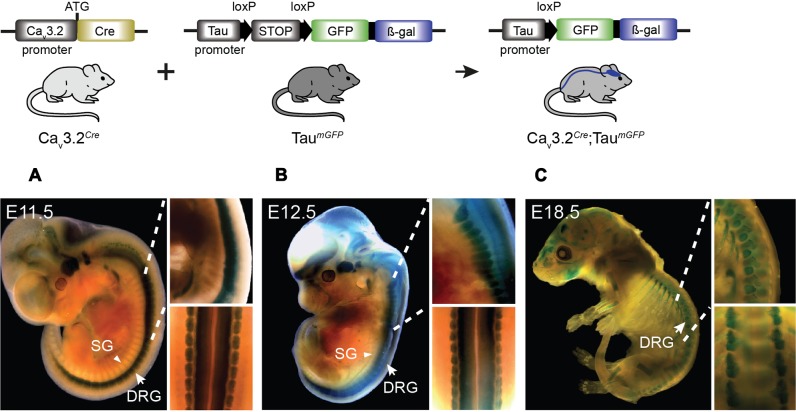
**Cre activation in Ca_v_3.2*^Cre^*; *Tau^mGFP^* mice in development.** β-gal expression in the nervous system of Ca_v_3.2*^Cre^*;*Tau^mGFP^* mice as shown by the blue staining. Embryos at stages **(A)** E11.5, **(B)** E12.5, and **(C)** E18.5 of development. Note the stronger β-gal staining in the spinal cord and DRGs in early stages compared to the brain. The staining observed in bones in the E18.5 embryos was also observed in negative controls. Zoom in panels: lateral and dorsal views of the spinal cord. DRG, dorsal root ganglia indicated with arrows; SG, sympathetic ganglia indicated with arrowheads.

**FIGURE 4:**
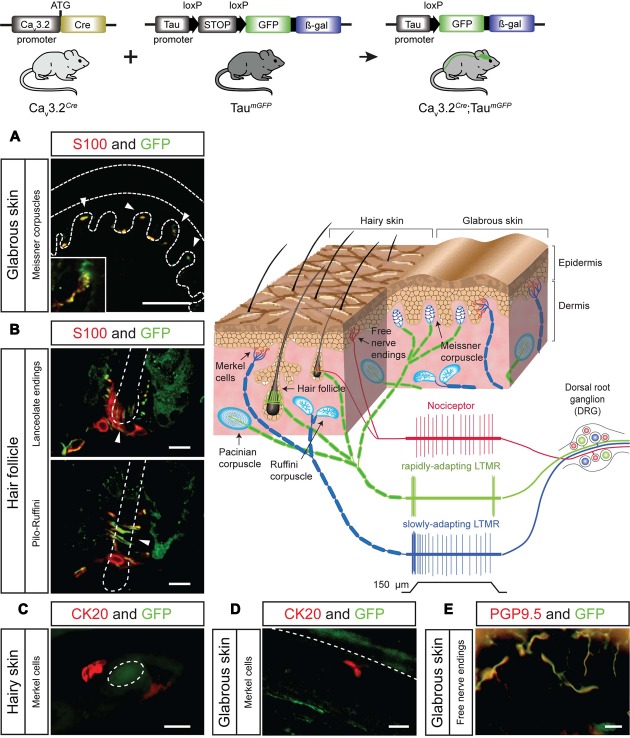
**Sensory end organs in the skin and their innervation by sensory afferents in Ca_v_3.2*^Cre^*;*Tau^mGFP^* mice. (A)** Co-immunostaining of GFP and S100 for visualization of Meissner’s corpuscles in sections of the hind paw glabrous skin. Dashed lines indicate the epidermis and dermis. Arrow heads indicate the location of the Meissner corpuscles. A magnification of one of the Meissner corpuscles is shown in the bottom left corner. Scale bar: 100 μm. **(B)** Co-immunostaining of GFP and S100 in hairy skin slices of the paw for visualization of lanceolate endings and pilo-Ruffini endings. Arrow heads indicate the structure of the nerve innervating the hair follicle. Scale bars: 20 μm. **(C,D)** Co-immunostaining of GFP and CK20 to visualize Merkel cells in hairy skin and in the glabrous skin slices. **(C)** Horizontal section of hairy skin. The dashed circle indicates the outline of a hair. Scale bar: 20 μm. **(D)** Perpendicular section of the glabrous skin of the paw. The dashed line indicates the border between epidermis and dermis. Scale bar: 20 μm. **(E)** Examples of GFP^+^ free nerve endings in the glabrous skin double-stained for the pan-neuronal marker PGP9.5. Scale bar: 20 μm. Cartoon: sensory end organs located in the skin innervated by primary afferent fiber endings. The response properties of nociceptors (red), rapidly adapting mechanoreceptors (green), and slowly adapting mechanoreceptors (green) to mechanical stimulation are illustrated.

The difference in the expression pattern of the Ca_v_3.2 channel inferred from Ca_v_3.2*^EGFP^* mice and the Ca_v_3.2*^Cre^* could be due to an early activation of the Ca_v_3.2 during development or to relatively weak Ca_v_3.2 promoter activity in many cells. We crossed the Ca_v_3.2*^Cre^* knock-in mouse with the Tau*^mGFP^* reporter line which is specific for the nervous system and analyzed the β-gal staining pattern in whole embryos at different stages of development. We observed positive β-gal staining in Ca_v_3.2*^Cre^*;Tau*^mGFP^* embryos as early as E11.5. At this stage, strong nuclear β-gal staining is detected in the hind brain, DRGs and spinal cord, and only a few positive cells were observed in the forebrain and midbrain (**Figure 3A** and zoom in). In addition, we noticed that there was no positive β-gal staining in the sympathetic ganglia (**Figure 3A**, arrow head). At E12.5, the number of β-gal positive cells in the same brain areas, DRGs and spinal cord had increased enormously (**Figure 3B** and zoom in), and remained constant until E18.5 (**Figure 3C** and zoom in). Thus, using this reporter line, we can demonstrate an early onset of Ca_v_3.2 promoter activity and that the Ca_v_3.2 ion channel is not expressed in the sympathetic ganglia at any point during development. One advantage of using the Tau*^mGFP^* reporter line is that mGFP is a membrane targeted protein and thus permits the tracing of axonal projections. This feature allowed us to analyze the innervation of the sensory end organs in the skin of Ca_v_3.2*^Cre^*;Tau*^mGFP^* animals. We used glabrous and hairy skin of the hindpaw of adult mice because of the diversity of end organs in these skin areas (Li et al., 2011; Heidenreich et al., 2012; Wende et al., 2012; Lechner and Lewin, 2013). Double immunofluorescence staining against mGFP and the Schwann cell marker S100 showed that Meissner’s corspuscles and hair follicles were innervated by mGFP^+^ afferents (**Figures 4A,B**). Meissner corpuscles are innervated by Aβ-myelinated fibers classified as rapidly adapting mechanoreceptors (RAMs) (Lumpkin and Caterina, 2007; Wende et al., 2012), and hair follicles can be innervated by Aβ- and/or Aδ-myelinated fibers that are also classified as RAMs or D-hair receptors (**Figure 4**, scheme) (Li et al., 2011; Lechner and Lewin, 2013). In addition, using cytokeratin 20 to visualize Merkel cells we found that mGFP^+^ fibers in Ca_v_3.2*^Cre^*;Tau*^mGFP^* animals were not associated with Merkel cells. We attempted to visualize mGFP^+^ fibers together with Merkel cells in three different mice and we could easily detect mGFP^+^ fibers associated with local hairs, but we never found such fibers in the Merkel disks of the hairy skin (**Figure 4C**). Touch domes are also found in the glabrous skin, but here as in hairy skin we found no evidence of mGFP^+^ fibers associated with these structures (**Figure 4D**). In the same skin regions we could always detect GFP free nerve endings, all double positive for the pan neuronal marker PGP9.5, which probably belongs to nociceptive afferent endings (**Figure 4E**). Merkel cells are innervated by Aβ-fibers with a slowly adapting (SA type I) response to mechanical stimuli (**Figure 4**, scheme) (Iggo and Muir, 1969). Thus, the lack of mGFP^+^ fibers in this end organ suggests that SA type I fibers arise from neural crest cells (NCCs) or progenitors that do not express the Ca_v_3.2 gene during development.

In the DRG, the majority of the neurons appear to be positive for mGFP in Ca_v_3.2*^Cre^*;Tau*^mGFP^* animals (Supplementary Figure 2), which is consistent with the great diversity of sensory endings that we observed in the skin. We further characterized the sensory neuron subtypes expressing mGFP in these animals using double immunofluorescence for other standard markers of sensory subtypes in the DRG. Many of the mGFP^+^ neurons were positive for NF200 suggesting that they contain myelinated Aβ- and Aδ-myelinated fibers, this is consistent with the innervation of hair follicles and Meissner’s corpuscles by mGFP^+^ afferents observed in the skin (Supplementary Figure 2A) (Bourane et al., 2009; Luo et al., 2009). We could also show that non-peptidergic nociceptors labeled with IB4, a marker for high-threshold C-unmyelinated fibers, were mGFP^+^ (Silverman and Kruger, 1990), as well as those sensory neurons positive for Tyrosine hydroxylase (TH), used to label putative low-threshold C-unmyelinated mechanoreceptors (C-LTMRs) (Supplementary Figure 2A) (Li et al., 2011). Thus, the data suggested that the Ca_v_3.2 ion channel was expressed in mechanoreceptors and nociceptors in the DRG. To confirm this, we sought to characterize the electrophysiological properties of these mGFP^+^ sensory neurons in cell culture.

An initial step to test the electrophysiological properties of these cells was to confirm that mGFP^+^ neurons could be visualized amongst cultures of DRG neurons isolated from Ca_v_3.2*^Cre^*;Tau*^mGFP^* mice (Supplementary Figures 2B,C). We took advantage of the strong mGFP^+^ signal to carry out an electrophysiological analysis of these neurons using the whole cell patch clamp technique. We examined mGFP^+^ cells 18 and 24 h post-isolation and recorded the AP in the current clamp mode after injection of current via the recording electrode. Increasing square wave pulses starting with 80 pA with a duration of 80 ms were used and such pulse protocols are sufficient to evoke APs is DRG neurons cultured from both wild type and Ca_v_3.2*^Cre^*;Tau*^mGFP^* mice (Wang and Lewin, 2011). We could classify the recorded mGFP^+^ neurons (*n* = 11) on the basis of their AP shape into putative mechanoreceptors or nociceptors. We measured the duration of the half peak amplitude, recovery time after hyperpolarization, and presence or absence of a hump on the falling phase of the AP. Our results highlighted the existence of two populations of cells with distinctive AP properties (Supplementary Figure 2B). Approximately 50% of the mGFP^+^ positive cells had narrow, humpless APs with short half peak duration and short hyperpolarization durations characteristic of mechanoreceptors (Djouhri et al., 1998; Petruska et al., 2000; Lawson, 2002; Lechner et al., 2009). The remaining 50% of the EGFP positive cells had APs with broad half peak durations and longer after-hyperpolarization durations characteristic of nociceptors (Supplementary Figure 2C).

**FIGURE 5:**
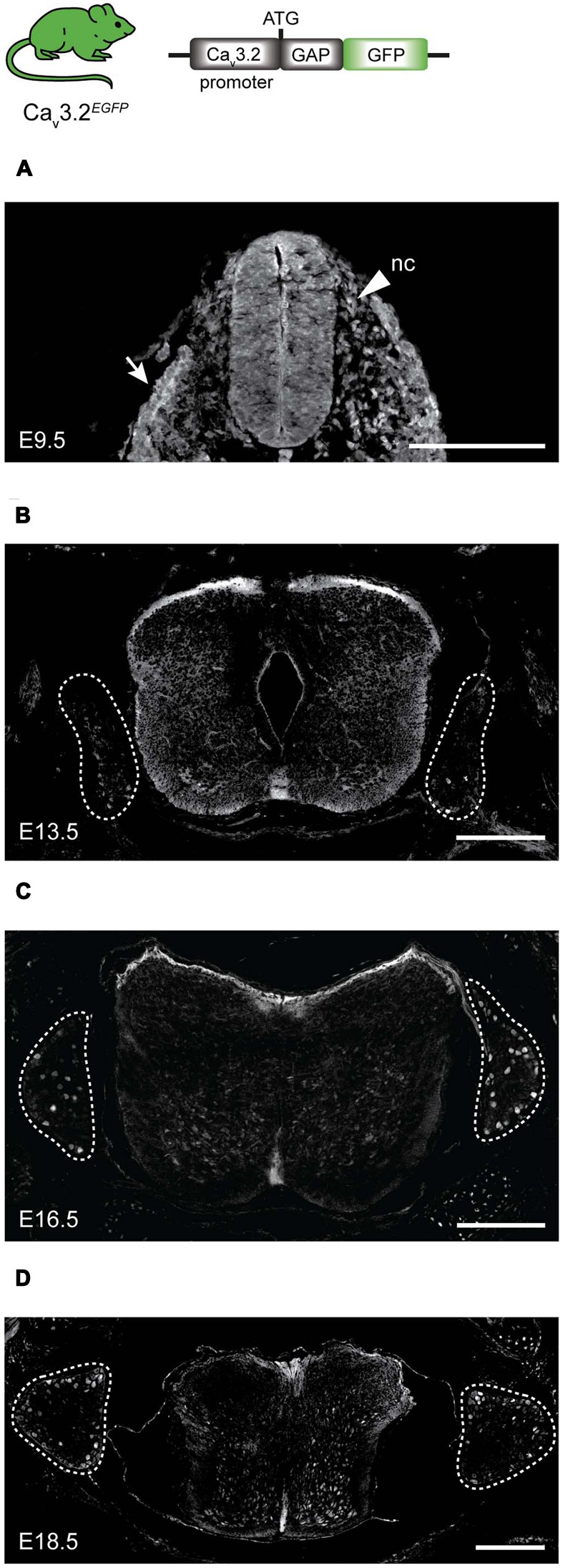
**EGFP expression during development in spinal cord and DRG of Ca_v_3.2*^EGFP^* knock-in mice. (A)** At E9.5, EGFP is expressed in some cells of the neuronal crest (nc) indicated by an arrow head, in the epithelium indicated by an arrow, and in the spinal cord. **(B)** At E13.5, a few cells of the DRG are EGFP^+^, while the staining at the dorsal side of the spinal cord is stronger. **(C)** At E16.5 the number of EGFP^+^ cells increases in the DRG, but staining in the spinal cord is reduced. **(D)** Finally at E18.5, EGFP expression in the DRG appears constant while the positive staining has almost disappeared in the spinal cord. Dashed lines outline the DRGs. Scale bars: 200 μm.

We could determine the onset of the Ca_v_3.2 gene promoter activity using the Ca_v_3.2*^Cre^*;Tau*^mGFP^* strain. However, this strain does not allow us to observe the dynamic activity of the promoter during development, for this reason we again used homozygotes Ca_v_3.2*^EGFP^* knock-in mouse to study the spatiotemporal pattern of the Ca_v_3.2 gene promoter in the spinal cord and DRG at different embryonic stages namely E9.5, E13.5, E16.5, and E18.5 (**Figure 5**). The EGFP protein could be detected in stages as early as E9.5 at the roof plate, in some cells at the dorsal ventricular zone of the spinal cord and in a fraction of the migrating NCCs that later form the DRG at E10.5. Positive EGFP cells were also observed in the epithelium (**Figure 5A**, white arrow). After the DRG formed at E13.5, EGFP expression was detected in a few cells within the ganglia, and a very strong fluorescent signal was observed in cells in the ventral spinal cord (**Figure 5B**). The number of EGFP^+^ cells in the DRG increased throughout developmental and reached approximately 7% at E16.5 (**Figure 5C**). The percentage of EGFP^+^ cells remained constant at E18.5 (**Figure 5D**). Surprisingly, we could not detect EGFP^+^ cells in the DRG and spinal cord of P0 Ca_v_3.2*^EGFP^* animals using antibodies against EGFP, but we could detect mRNA expression at this postnatal stage using qPCR (data not shown). However, very shortly before birth at E18.5 we found that around 7% of the DRG cells were GFP^+^ in Ca_v_3.2*^EGFP^* knock-in mice. The percentage of EGFP^+^ cells in the DRG at E18.5 matches well with the reported number of cells shown to express Ca_v_3.2 mRNA in the adult mouse DRG (Talley et al., 1999; Shin et al., 2003). However, our data indicate fewer Ca_v_3.2 positive neurons at E18.5 than detected using a recently published Ca_v_3.2 knock-in mouse in which a EGFP sequence was inserted within the Ca_v_3.2 protein coding sequence (François et al., 2015).

We decided to characterize the molecular features of the small population of EGFP^+^ neurons observed in the DRG at E18.5 in our Ca_v_3.2*^EGFP^* knock-in mice (Supplementary Figure 3). Co-immunostaining analyses of EGFP and molecular markers for sensory neuron subtypes such as the Trk receptors, revealed that about 45 ± 13% of the EGFP^+^ cells are TrkB^+^, which has already been shown to be a good marker of D-hair receptors (Shin et al., 2003; Li et al., 2011). However, not all TrkB^+^ were positive for EGFP, 55 ± 2.8% of the EGFP^+^ neurons were found to be TrkC^+^, and only 13 ± 4.1% were TrkA^+^ (Supplementary Figures 3A,B). We did not observe co-labeling of EGFP with IB4 (data not shown), indicating that non-peptidergic nociceptors do not express Ca_v_3.2 (Silverman and Kruger, 1990; Molliver et al., 1997; Stucky and Lewin, 1999), and 28 ± 19% of the EGFP^+^ neurons were putative C-LTMRs (data not shown) as observed by co-labeling with a TH antibody (Li et al., 2011).

**FIGURE 6:**
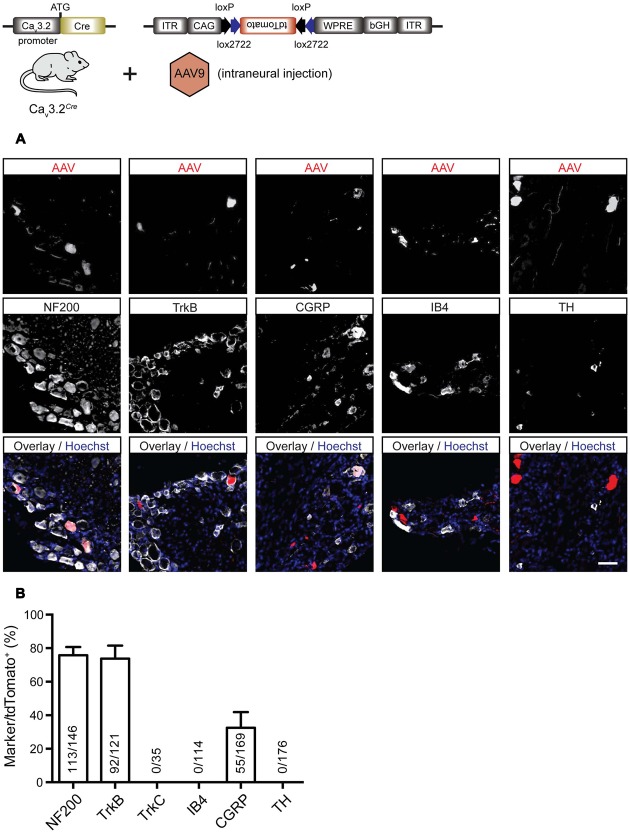
**Immunostainings for characterization of tdTomato positive cells in the DRG of virally transduced Ca_v_3.2*^Cre^* mice. (A)** Immunostainings of tdTomato^+^ cells with markers for sensory neuron subtypes, i.e., NF200, TrkB, TrkC, CGRP, IB4, and TH. Scale bar: 50 μm. **(B)** Quantification of double labeled cells. The ordinate represents tdTomato^+^ cells co-expressing one of the molecular markers used divided by the total number of tdTomato^+^ cells in the DRG. Data presented as mean + SD. *N* = 3 animals, at least three DRGs per animal were examined.

In order to characterize more precisely the sensory neurons in the adult DRG that express Ca_v_3.2 in the mature somatosensory system we used a viral reporter approach. The AAV-flex-tdTomato particles were injected into the sciatic nerve of adult heterozygous Ca_v_3.2*^Cre^* mice. Four to eight weeks after viral infection we obtained robust and intense labeling of a small population of sensory neurons with tdTomato in the lumbar DRGs. We performed a molecular analysis of this population by co-labeling these cells with various molecular markers like NF200, TrkB, TrkC, IB4, calcitonin-gene related peptide (CGRP), and TH. The vast majority (77 ± 4.3%) of tdTomato^+^ cells expressed NF200 and also 76_-_ ± 6.9% of virally transduced Ca_v_3.2*^Cre^* cells were TrkB^+^. A smaller percentage of tdTomato^+^ cells were found to be CGRP^+^ (33 ± 6.6%) (**Figures 6A,B**). No co-labeling of tdTomato was found with TrkC (images not shown) or TH (**Figures 6A,B**). We also never observed IB4-positive tdTomato^+^ neurons. Control experiments in which AAV-flex-tdTomato was injected into the sciatic nerve of adult Advillin*^Cre^* mice (Zurborg et al., 2011) revealed that all examined neuronal subtypes but TH^+^ cells expressed tdTomato (Supplementary Figure 4). This result suggest that the AAV serotype 9 does not transduce TH^+^ primary sensory neurons. Another possibility could be that TH^+^ neurons do not express Advillin since Advillin*^Cre^* expression has been shown not to be present in up to 18% of DRG neurons (Zurborg et al., 2011).

**FIGURE 7:**
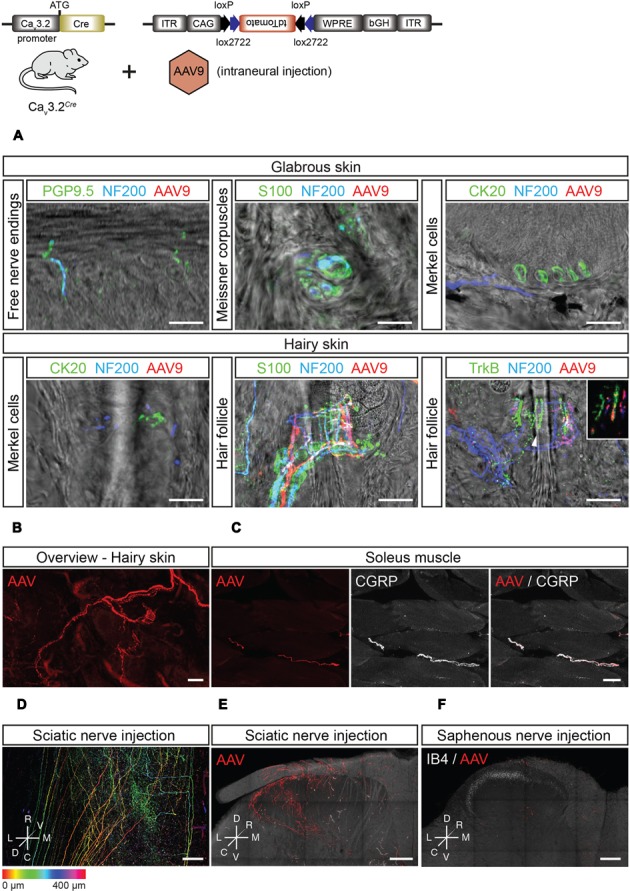
**Peripheral and central innervation of tdTomato positive cells of virally transduced Ca_v_3.2*^Cre^* mice. (A)** Sensory end organs in the skin and their innervation by sensory afferents in virally transduced Ca_v_3.2*^Cre^* mice after intrasciatic AAV injection. Sensory fibers were labeled using NF200 and/or PGP9.5. In the glabrous skin, Meissner corpuscles were visualized using an antibody against S100, and Merkel cells were stained using an antibody against CK20. In the hairy skin, Merkel cells were also immunostained using an antibody against CK20. Hair follicles were visualized by immunostaining of terminal Schwann cells using an antibody against S100. Hair follicle afferents were immunostained with antibodies against NF200 and TrkB. Scale bars: 20 μm. **(B)** Whole-mount imaging of hairy skin with tdTomato^+^ fibers innervating hair follicles. Scale bar: 25 μm. **(C)** In the soleus muscle, tdTomato^+^ sensory afferents were observed and identified as nociceptors by immunostaining using an antibody against CGRP. Scale bar: 20 μm. **(D–F)** Central termination of virally transduced Ca_v_3.2*^Cre^* positive fibers after intrasciatic AAV injection. **(D)** Dorsoventral projection (with depth color coding) of a tiled image stack of the lumbar spinal cord of a Ca_v_3.2*^Cre^* mouse showing TdTomato^+^ central terminals of Ca_v_3.2*^Cre^* expressing sensory neurons, Scale bar: 100 μm. **(E)** Maximum intensity projections of a thick transverse slice of the lumbar spinal cord after intra-sciatic nerve AAV injection. Scale bar: 100 μm. **(F)** Maximum intensity projection of a thick transverse slice of the lumbar spinal cord after intra-saphenous nerve AAV injection. Scale bar: 100 μm.

Viral-based gene transfer proved to be a robust way to visualize the sensory-endings of Ca_v_3.2 expressing DRG neurons in both the hairy and glabrous skin (**Figures 7A,B**). In the glabrous skin, there were no tdTomato^+^ fibers associated with free nerve endings, Meissner’s corpuscles or Merkel cells (**Figure 7A**). Similarly, in hairy skin, neither free nerve endings (data not shown) nor sensory fiber endings innervating Merkel cells were tdTomato^+^ (**Figure 7A**). However, tdTomato^+^ lanceolate endings, but not circumferential endings, were found surrounding hair follicles which were co-labeled with antibodies against NF200 and TrkB (**Figures 7A,B**).

Interestingly, we could also visualize the central projections of Ca_v_3.2 expressing DRG neurons in the spinal cord dorsal horn after intraneural AAV-flex-tdTomato injection (**Figures 7D–F**). Sciatic nerve injections of AAV-flex-tdTomato injections resulted in labeling of fiber terminals in the superficial and deep dorsal horn laminae. It is known that D-hair mechanoreceptor afferents terminate exclusively within the deep dorsal horn with typical flame shaped arbor with terminals not penetrating into lamina II (Light and Perl, 1979). However, genetic tracing experiments using a TrkB*^tauEGFP^* mouse have suggested penetration of terminals into lamina II (Li et al., 2011). The projection to the deep dorsal horn found after intraneural injection of the virus was consistent with a specific labeling of D-hair mechanoreceptors which show a highly restricted medio-lateral distribution that reflects the somatotopy of the sciatic nerve projection. In contrast, the medio-laterally broad projection of tdTomato^+^ fibers in the superficial dorsal horn labeled from the sciatic nerve is not consistent with a projection from D-hair mechanoreceptors. This projection is reminiscent of the projection of singly labeled nociceptors from deep tissue (Ling et al., 2003) and this population may represent the peptidergic nociceptors found to be double positive for CGRP and tdTomato in the ganglia (**Figures 6A,B**). Considering that in the skin, no tdTomato^+^ free nerve endings were found, we hypothesized that the lamina I projection must arise from CGRP^+^ fibers innervating muscle. Indeed in Ca_v_3.2*^Cre^* animals in which the AAV-flex-tdTomato virus was injected into the sciatic nerve we could detect GGRP^+^ fibers within the gastrocnemius muscle (**Figure 7C**). To further test this idea, we performed AAV-flex-tdTomato injections into the saphenous nerve which contains only cutaneous afferents as opposed to the sciatic nerve which is a mixed nerve. Injection of AAV-flex-tdTomato into the saphenous nerve did not result in labeling of fibers terminating in lamina I but only in labeling of fibers projecting to deeper dorsal horn with morphologies typical of D-hair mechanoreceptors (**Figure 7F**). Thus, it can be concluded that CGRP^+^ Ca_v_3.2 expressing neurons innervate muscle or joint, but not the hairy skin.

T-type calcium currents have been recorded in spinal interneurons and deep dorsal horn neurons (Ikeda, 2003; Rivera-Arconada and Lopez-Garcia, 2015) and so we also asked whether the Ca_v_3.2*^Cre^* promoter was also active in the spinal cord. We made intrathecal injections of AAV-flex-tdTomato in adult heterozygous Ca_v_3.2*^Cre^* mice which resulted in labeling of many dorsal horn and ventral horn neurons (Supplementary Figures 5A,B). Thus, Ca_v_3.2*^Cre^* mice appear to be a useful tool to analyze specific populations of spinal neurons that express the Ca_v_3.2 ion channel in the adult.

## Discussion

Here, we describe the generation and characterization of two knock-in mouse strains to accurately determine the temporal and spatial expression pattern of the Ca_v_3.2 channel gene in the mouse nervous system. By using antibody detection of EGFP in the Ca_v_3.2*^EGFP^* mouse, and crossing the Ca_v_3.2*^Cre^* strain with the Tau*^mGFP^* or the Rosa26*^LacZ^* mouse reporter lines, we show that the Ca_v_3.2 gene is mainly expressed in the DG of the adult brain. We have, however, concentrated our analysis on the expression of the Ca_v_3.2 gene in the somatosensory system as many studies have shown a role for this important calcium channel in regulating sensory neuron excitability as well as the gating of sensory information in the spinal cord (Ikeda, 2003; Shin et al., 2003; Bourinet et al., 2005; Heppenstall and Lewin, 2006). Using our two new mouse models we were able to identify sensory neuron progenitors expressing the Ca_v_3.2 channel gene during development. We show that NCCs expressing the Ca_v_3.2 channel at E9.5 specifically give rise to a large population (∼90%) of DRG neurons (Supplementary Figure 2A). However, NCCs giving rise to sympathetic neurons and a specific population of SAMs that innervate Merkel cells in the skin never express the Ca_v_3.2 channel gene. Using our Ca_v_3.2*^EGFP^* we could show that a much smaller population of DRG neurons express the Ca_v_3.2 gene in late embryonic development. Thus, we observed that the Cav3.2 channel is expressed in about 7% of the DRG cells, which were mainly TrkB^+^ and TrkC^+^, with only a small percentage expressing TrkA. In a further analysis using viruses containing a Cre-dependent reporter in our Ca_v_3.2*^Cre^* mice we could label and characterize the nature of adult sensory neurons that express Ca_v_3.2. The results of these experiments revealed that there are two major populations of Ca_v_3.2 expressing sensory neurons; first low threshold Aδ-fiber mechanoreceptors (D-hair receptors) that exclusively innervate hair follicles and a population of peptidergic nociceptors that innervate muscle, but not the skin. We could not demonstrate expression of Ca_v_3.2 in tyrosine hydroxylase positive sensory neurons which have been postulated to be C-fiber low threshold mechanoreceptors. It is not clear at this time whether the failure to label putative C-LTMRs was due to technical limitations of the AAV virus reporter approach.

In the brain, the Ca_v_3.2 channel shapes neuronal firing patterns of hippocampal and thalamic neurons (Crunelli et al., 2005; Cain and Snutch, 2010; Deleuze et al., 2012; Tringham et al., 2012). Strong expression of the Ca_v_3.2 gene has been reported in neurons of the CA1, CA3 fields, and in granule cells of the hippocampus (Talley et al., 1999; McKay et al., 2006). Also, expression of the Ca_v_3.2 and Ca_v_3.3 subunit genes has been observed in the reticular thalamic nucleus (RT) (Talley et al., 1999; McKay et al., 2006), and here Ca_v_3.2 has been proposed to modulate tonic firing regularity and spike frequency during burst firing (Liao et al., 2011). Using our Ca_v_3.2*^EGFP^* knock-in mice we could show that EGFP was predominantly expressed in the DG, and in the internal capsule of the brain. Surprisingly, we did not observe activation of the Ca_v_3.2 promoter in the RT of the Ca_v_3.2*^EGFP^* or the Ca_v_3.2*^Cre^* mouse, even though the Ca_v_3.2*^Cre^* knock-in mouse allowed us to determine promoter activity in brain regions where the Ca_v_3.2 channel was poorly expressed. Instead, we detected activation of the Ca_v_3.2 promoter in the lateral-dorsal nucleus of the thalamus (**Figure 2A**). Nevertheless, the CNS expression data from our reporter mice broadly agrees with those found using conventional *in situ* hybridization techniques (Talley et al., 1999).

In the developing somatosensory system, Ca_v_3.2 gene expression has a very early onset and displays a highly dynamic spatiotemporal expression pattern. In Ca_v_3.2*^EGFP^* embryos, EGFP expression was already observed in some of the trunk NCCs at E9.5. However, since NCCs give rise to a wide variety of cell types including sensory neuron lineages, sympathetic neurons, melanocytes, and Schwann cells (Serbedzija et al., 1990; Jessen and Mirsky, 2005; Takahashi et al., 2013), it was interesting to observe that in Ca_v_3.2*^Cre^*;Tau*^mGFP^* embryos, β-gal activity was restricted to DRG neurons and was not found in sympathetic neurons. Non-neuronal cell populations generated from NCCs such as Schwann cells, satellite cells, and melanocytes are expected not to be labeled in Ca_v_3.2*^Cre^*;Tau*^mGFP^* embryos (Opdecamp et al., 1997; Jessen and Mirsky, 2005; Takahashi et al., 2013), but we also did not find evidence of EGFP positive glial cells in sections from the Ca_v_3.2*^EGFP^* embryos.

At E13.5, when the three waves of DRG neurogenesis are complete (Ma et al., 1999), Ca_v_3.2 promoter activity was detected in only a few sensory neurons in the DRG of Ca_v_3.2*^EGFP^* embryos. At E16.5 the number of EGFP^+^ cells was found to be around 7% of the total DRG and appeared to remain constant until E18.5. The steady and unchanging expression of the Ca_v_3.2 promoter suggests that the transcriptional regulation of the Ca_v_3.2 channel is a result of early sub-type specification. Indeed, E16.5 sensory neurons with the electrophysiological properties of mechanoreceptors appear already mature at this stage (Lechner et al., 2009), consistent with the idea that Ca_v_3.2 channel is an early marker of this population.

Characterization of the Ca_v_3.2-positive sub-population in Ca_v_3.2*^EGFP^* embryos at E18.5 revealed that the majority of the EGFP^+^ cells at this stage express TrkB and TrkC, and only a low percentage of them express TrkA. There is high probability that EGFP^+^/TrkB^+^ and EGFP^+^/TrkC^+^ cells in the DRG developed from the early TrkB/C sensory linage expressing Runx3/Shox2 transcription factors (Levanon et al., 2002; Kramer et al., 2006; Scott et al., 2011), and not from the early Ret (eRet^+^/MafA^+^) population (Bourane et al., 2009; Luo et al., 2009). This idea is supported by a recent study on the transcription factor cMaf in sensory neurons where cMaf acts upstream of MafA, and loss of function alleles cause specific alterations in the physiological properties of Aβ-fiber mechanoreceptors without effects on Aδ-fibers. Interestingly, the development of Ca_v_3.2 positive sensory neurons was unchanged in cMaf mutants (Wende et al., 2012). TrkA^+^ DRG cells develop in two waves, the first wave gives rise to Aδ-fibers and is generated from the Ngn2-dependent precursor pool that are Cux2-positive (Bachy et al., 2011). The second wave is generated from the Ngn1-dependent wave of precursors which are Cux2-negative (Averill et al., 1995). Because the EGFP^+^/TrkA^+^ cells were IB4-negative, and previous studies had shown that the Ca_v_3.2 channel is expressed in medium size DRG cells (Shin et al., 2003), it may be that these EGFP^+^/TrkA^+^ neurons developed from the Ngn2-dependent wave of neurogenesis.

The function of the Ca_v_3.2 channel in undifferentiated DRG precursor cells is unclear. However, it has been reported that LVA Ca^2+^, but not HVA Ca^2+^ currents can be recorded in undifferentiated precursor cells of the chick DRG, while both conductances are observed in differentiated sensory neurons (Gottmann and Lux, 1990; Gottmann et al., 1991). Our study definitively identifies the Ca_v_3.2 channel as one of the T-type α-subunits expressed in sensory precursor cells. Loss of function alleles of the Ca_v_3.2 gene have not, however, been reported to affect the specification of sensory lineages (Wang and Lewin, 2011).

Expression of the Ca_v_3.2 channel has been observed in small to medium size DRG and has been proposed as a molecular marker for D-hair receptors (Shin et al., 2003; Dubreuil et al., 2004; Aptel et al., 2007; François et al., 2015). In addition, the Ca_v_3.2 channel has been shown to be required for the normal temporal coding of moving stimuli by D-hair receptors (Wang and Lewin, 2011). Thus, the presence of EGFP^+^/TrkB^+^ DRG neurons was expected since signaling of NT-4 through TrkB is required for D-hairs survival in mature mice (Stucky et al., 1998, 2002). Expression of TrkB has been observed in D-hair receptors by Shin et al. (2003), and more recently, this receptor has been proposed as a molecular marker for D-hair receptors (Li et al., 2011). In that study, Li et al. (2011) generated the TrkB*^tauEGFP^* knock-in mouse to visualize TrkB expressing cells in the DRG. They showed that in thoracic DRG from TrkB*^tauEGFP^* mice, EGFP^+^ cells were medium size neurons, did not express the TH, IB4 or c-Ret, and in hairy skin they innervated longitudinal lanceolate endings associated with zigzag and awl/auchene hair follicles, and electrophysiological characterization classified them as D-hair receptors (Li et al., 2011). Using a Cre-dependent viral reporter approach, we now show that our Ca_v_3.2*^Cre^* mice can be used to label a highly specific population of cutaneous receptors that are identical to TrkB^+^ D-hair mechanoreceptors. Thus, infection of sensory axons with a AAV-flex-tdTomato virus labeled medium sized sensory neurons that exclusively make lanceolate endings on hair follicles in the hairy skin, glabrous skin was completely free of any innervation from Ca_v_3.2^+^ endings (**Figures 7A,B**). What was even more striking was that these Ca_v_3.2^+^ fibers also appeared to provide a specific and organized central projection into the deep dorsal horn as expected from previous work (Light and Perl, 1979) (**Figures 7D–F**).

Previous studies do not support the idea that TrkB is found uniquely in Aδ-fibers. Indeed, in the mouse there is a subpopulation of Aβ-fiber RA low-threshold mechanoreceptors that innervate Meissner corpuscles and/or lanceolate endings which are TrkB^+^/MafA^+^/cRet^+^ (Bourane et al., 2009; Luo et al., 2009; Wende et al., 2012). Also, lack of BDNF, the other TrkB high affinity ligand, affects the mechanical threshold of SA-mechanoreceptors, suggesting that TrkB receptors are also expressed in SA-mechanoreceptors innervating Merkel cells and/or Ruffini corpuscles (Carroll et al., 1998). In contrast we show here that the use of our Ca_v_3.2*^Cre^* in combination with viral reporters injected into cutaneous nerves provides a remarkably specific method to label and manipulate D-Hair mechanoreceptors.

The Ca_v_3.2 channel has also been proposed to play a role in nociception (Todorovic et al., 2001; Bourinet et al., 2005; Nelson et al., 2005; Choi et al., 2006). However, our Ca_v_3.2*^EGFP^* knock-in mouse shows that only a small percentage of EGFP^+^ DRG cells could be classified as peptidergic nociceptors since they express TrkA and are IB4-negative. We have now identified the nature of the Ca_v_3.2^+^ nociceptive population within the DRG that have molecular properties of nociceptors and these CGRP^+^ neurons innervate muscle (**Figure 7C**) and have projections in superficial laminae that are typical for muscle nociceptors (Ling et al., 2003) (**Figure 7E**). Recently, it has been proposed that another sensory neuron type with unmyelinated C-fiber axons the so-called C-LTMR may also express Ca_v_3.2 (François et al., 2015; Reynders et al., 2015). This sensory neuron population have been shown to be TH^+^ (Li et al., 2011), but we were not able to observe sensory neurons in Ca_v_3.2*^Cre^* infected with reporter constructs that were double positive for TH and dtTomato (**Figure 6**). It is not clear whether our failure to detect Ca_v_3.2^+^ sensory neurons with molecular features of C-LTMRs is due to an inability of the AAV9 virus to infect TH^+^ cells.

The reported changes in pain behavior after ablation of Ca_v_3.2 channels in sensory neurons should now been seen in the context that a substantial number of sensory neurons innervating the muscle express this channel. The importance of muscle nociceptors in chronic pain has often been underestimated although activation of such neurons can provoke powerful central sensitization (McMahon et al., 1993; Lewin et al., 2014). We also injected our reporter virus centrally and observed a substantial number of labeled dorsal and ventral horn interneurons in Ca_v_3.2*^Cre^* mice (Supplementary Figure 5). This finding emphasizes the fact that pharmacological or genetic manipulation of Ca_v_3.2 channels may modulate pain behavior by acting at sites within the spinal cord. The tools we have developed here should make possible a more detailed dissection of the role of such interneurons in pain and hyperalgesia.

In summary, our genetic models provide a comprehensive picture of Ca_v_3.2 channel expression in the developing and mature nervous system. Our study shows that the Ca_v_3.2 gene is active in early, committed migrating NCCs that give rise to a restrictive lineage of sensory neurons, but not sympathetic neurons. The expression of Ca_v_3.2 gene is rapidly restricted to early post-mitotic neurons some of which are identical to D-hair low-threshold mechanoreceptors already at E13.5. However, we also show that a small population of muscle nociceptors also express Ca_v_3.2 channels in the adult DRG. Ca_v_3.2 is the only ion channel so far identified whose expression in the neural crest defines a sub-population of committed sensory precursors.

## Author Contributions

YABS and AK designed the knock-in constructs. YABS generated knock-in animals, performed crossings with reporter lines, immunostainings and electrophysiological characterization. JH carried out viral injection, immunostainings and two-photon microscopy. VB helped YABS in generating Ca_v_3.2 reporter lines. YABS, JH, and GL planned experiments and analyzed data. YABS, JH, and GL wrote the paper.

## Conflict of Interest Statement

The authors declare that the research was conducted in the absence of any commercial or financial relationships that could be construed as a potential conflict of interest.
