# Supplementary material for: Genetic Tracing of Cav3.2 T-Type Calcium Channel Expression in the Peripheral Nervous System
Source: Front Mol Neurosci. 2017 Mar 15;10:70. doi: 10.3389/fnmol.2017.00070 (PMC5350092; doi:10.3389/fnmol.2017.00070)
Supplement: Supplementary file 1 [file Data_Sheet_1.docx]

Supplementary Material

Genetic tracing of Ca_v_3.2 T-type calcium channel expression in the peripheral nervous system

Yinth Andrea Bernal Sierra, Julia Haseleu, Alexey Kozlenkov, Valérie Bégay, Gary R. Lewin

*** Correspondence:** Gary R. Lewin: glewin@mdc-berlin.de

# Supplementary Figures

## Supplementary Figure 1


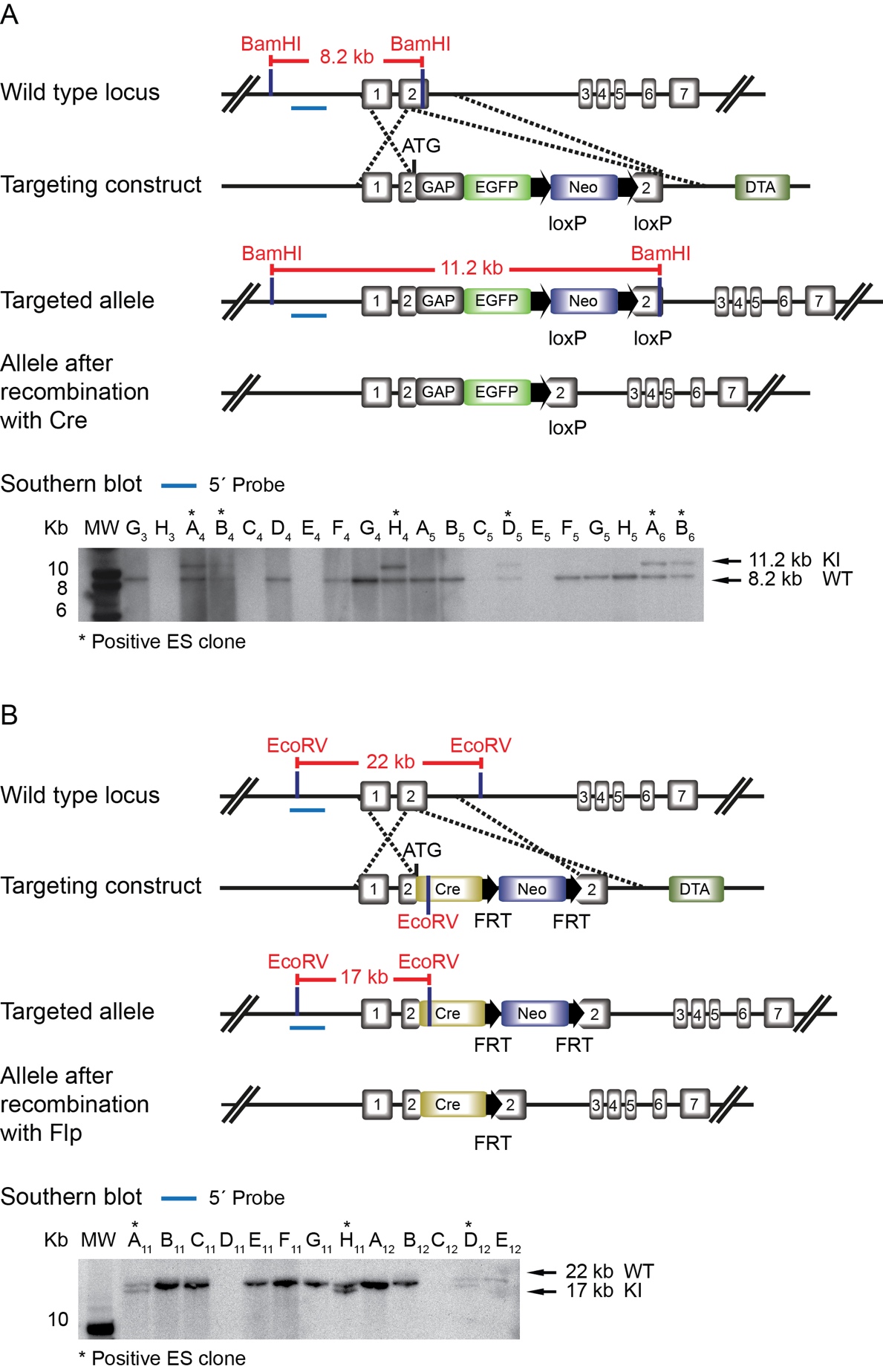


**Supplementary Figure 1. Generation of Ca_v_3.2*^eGFP^* and Ca_v_3.2*^Cre^* knockin mice.** **(A)** Schematic representation of the Ca_v_3.2 locus, the targeting vector and the mutated Ca_v_3.2*^EGFP^* allele. In the targeting vector, the EGFP cassette containing the palmitoylation site of GAP43 (GAP) is inserted after the ATG initiation codon located in the second exon of the Ca_v_3.2 gene, and is followed by a neomycin (Neo) cassette flanked by loxP sequences. A MC-1 diphteria toxin A (DTA) cassette located at the 3’ end of the vector was used for negative selection. At the bottom of **(A)**, a Southern blot analysis of *BamH*1 digested tail genomic DNA is shown. The 5’ end probe (blue line) shows a 8.2 kb band (red line) from the wild type locus and a 11.2 kb band (red line) from the targeted allele. *, Ca_v_3.2*^EGFP^* positive clone. **(B)** Schematic representation of the Ca_v_3.2 locus, the targeting vector, and the mutated Ca_v_3.2^Cre^ allele. In the targeting vector, a Cre recombinase cassette followed by a FRT-flanked Neo cassette is inserted in the second exon as described above. A DTA cassette was inserted at the 3’ end of the vector and was used for negative selection. At the bottom of **(B),** a Southern Blot analysis of *EcoR*V digested tail genomic DNA is shown. The 5’ end probe (blue line) shows a 22 kb band (red line) from the wild type locus and a 17 kb band (red line) from the targeted allele. *, Ca_v_3.2*^Cre^* positive clone. Note that the schemes are not in scale.

**Supplementary Figure 2**

**
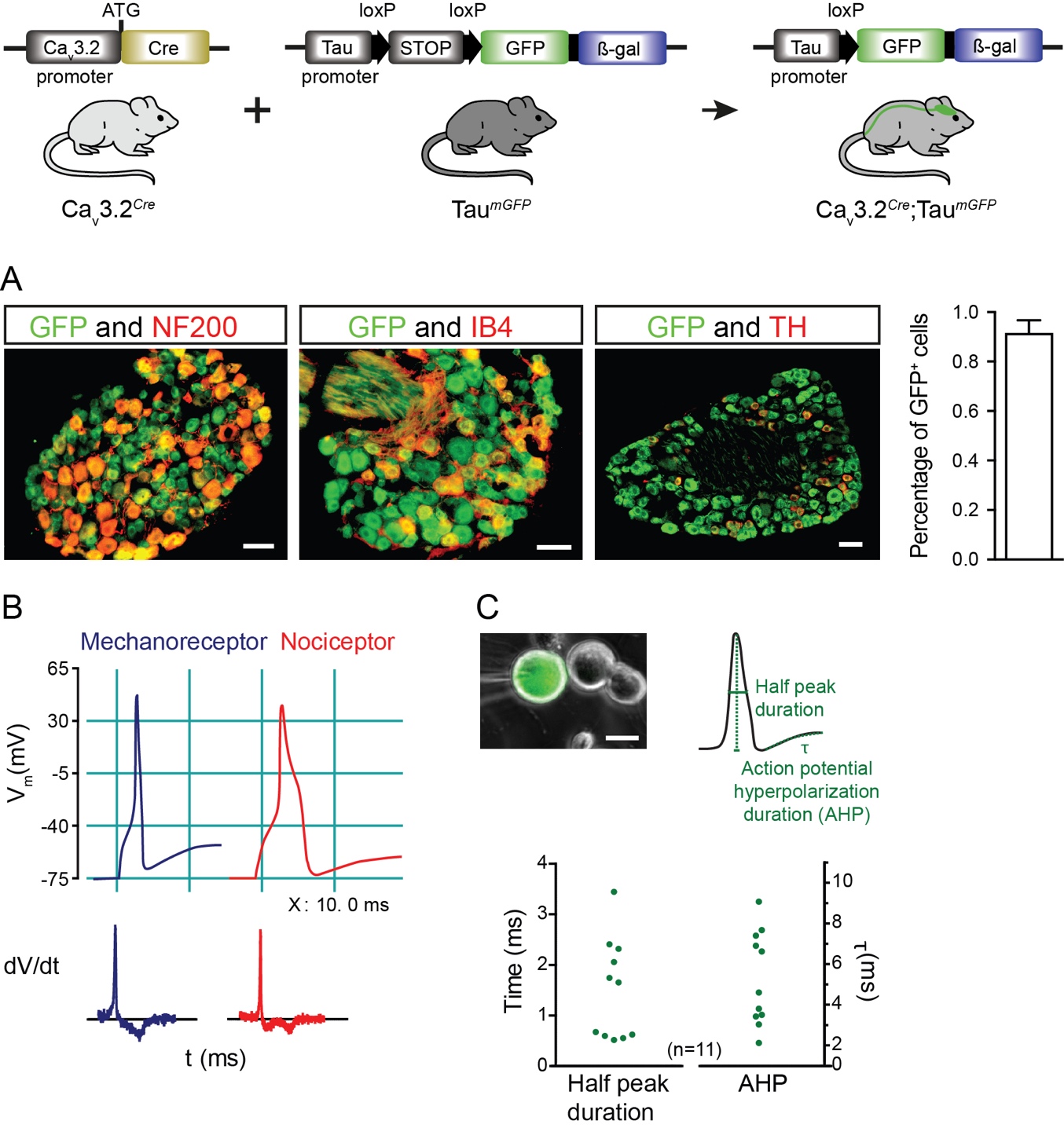
**

**Supplementary Figure 2. Characterization of GFP positive cells in the DRG of Ca_v_3.2*^Cre^*; *Tau^mGFP^* adult mice**. **(A)** Double immunostaining of GFP and NF200 for identification of Aß- and Aδ-myelinated afferents, GFP and IB4 for identification of nonpeptidergic unmyelinated nociceptors, and GFP and TH for identification of nonpeptidergic C-LTMRs. On the left, a quantification of GFP^+^ cells is shown. Scale bars: 50 µm. **(B)** Examples of the action potential shape of mechanoreceptors and nociceptors. Below, schemes of the first derivate dV/dt which show the presence of one minimum for mechanoreceptors and two minimums for nociceptors are shown. **(C)** In the upper left, an example of a GFP^+^ cell in culture is shown. Scale bar: 20 µm. In the upper right, an illustration of the measured action potential variables is shown. In the dotplot graph, electrophysiological parameters of action potentials recorded in cultured GFP^+^ DRG cells from Ca_v_3.2*^Cre^*;*Tau^mGFP^* mice are quantified. Cells with a half peak duration of less than 1 ms were classified as mechanoreceptors.

**Supplementary Figure 3**


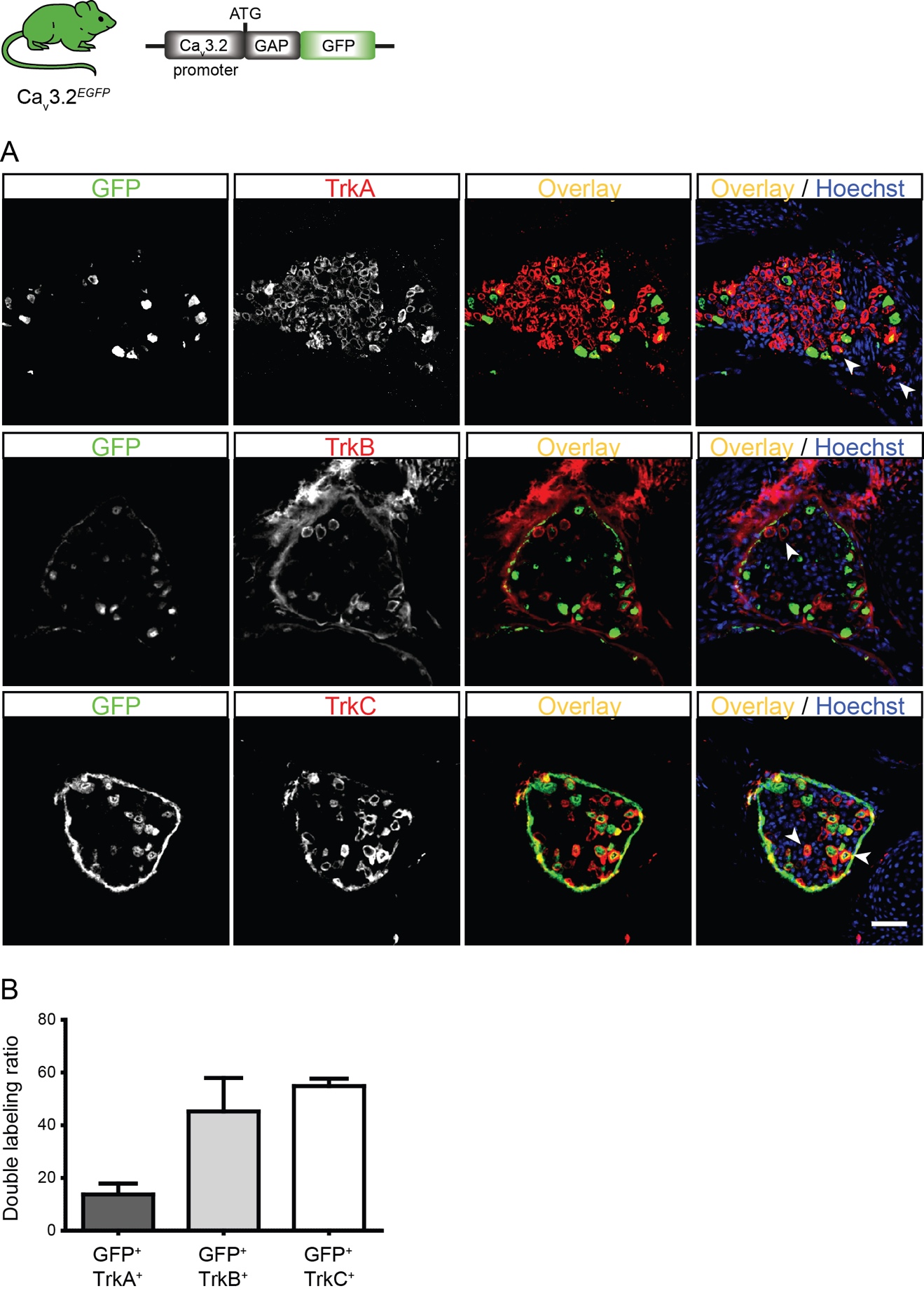


**Supplementary Figure 3. Characterization of eGFP^+^ cells in the DRG of Ca_v_3.2*^eGFP^* knockin mice.** **(A)** DRG double immunostaining of eGFP and TrkA, eGFP and TrkB, and eGFP and TrkC in embryos at E18.5. Cell nuclei are labelled with Hoechst (blue). Arrowheads indicate examples of double-positive neurons. Scale bars: 50 µm. **(B)** In the bar plot double immunostainings are quantified. The ordinate represents the percentage of eGFP^+^ cells co-expressing one of the molecular markers. Data presented as mean + SD. N=3 animals, 3 DRGs were examined per animal.

**Supplementary Figure 4**

**
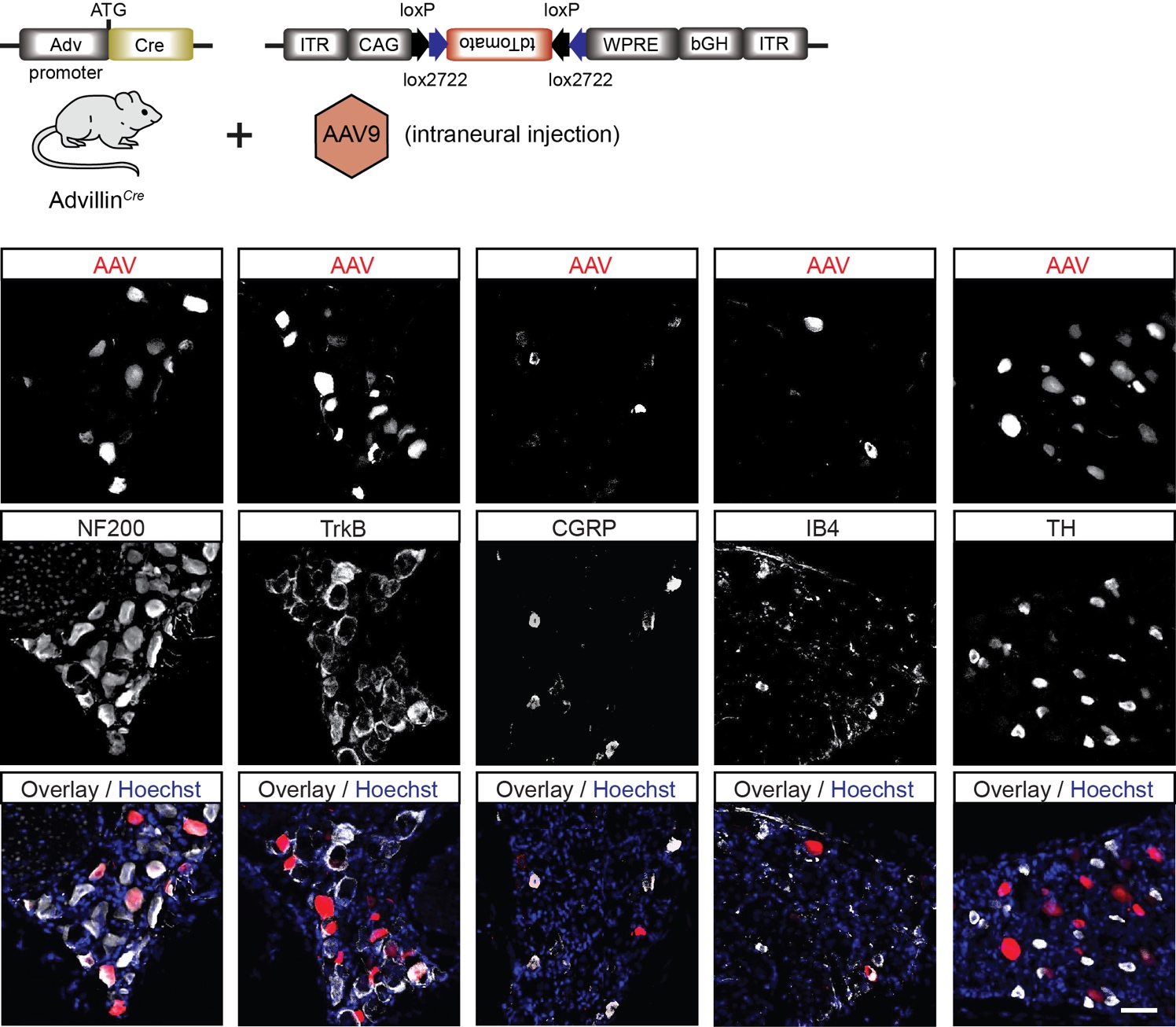
**

**Supplementary Figure 4. Immunostainings for characterization of tdTomato positive cells in the DRG of virally transduced Advillin*^Cre^* mice.** Representative images of immunostainings of tdTomato^+^ cells with markers for sensory neuron subtypes, i.e. NF200, TrkB, CGRP, IB4, and TH. Scale bar: 50 µm.

**Supplementary Figure 5**


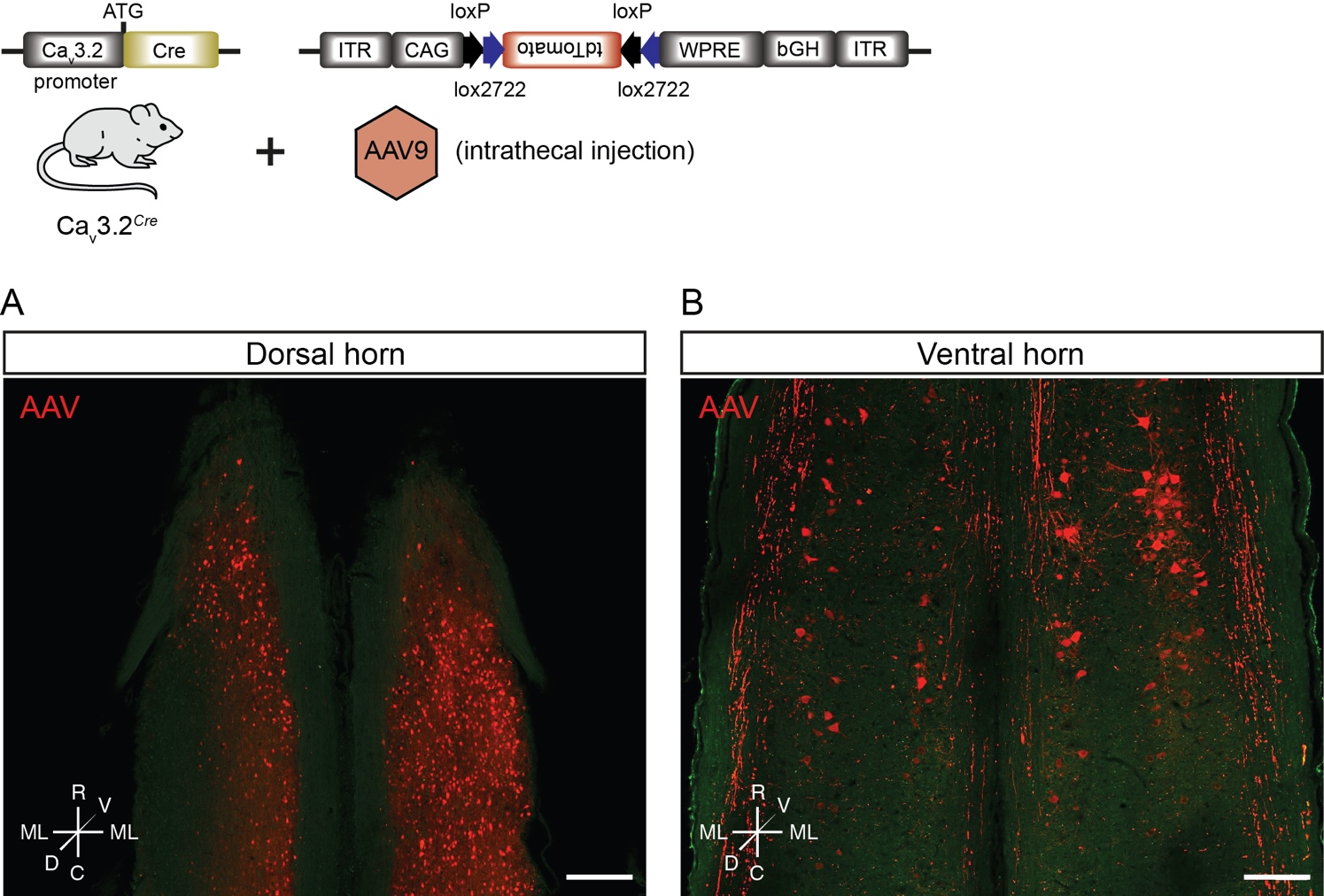


**Supplementary Figure 5. Virally transduced spinal cord cells in Ca_v_3.2*^Cre^* mice after intrathecal AAV injections.** TdTomato^+^ cells in the spinal cord **(A)** dorsal horn and **(B)** ventral horn. Scale bar: 200 µm.
